# Supplementary material for: Glass and Jamming Rheology in Soft Particles Made of PNIPAM and Polyacrylic Acid
Source: Int J Mol Sci. 2021 Apr 14;22(8):4032. doi: 10.3390/ijms22084032 (PMC8070831; doi:10.3390/ijms22084032)
Supplement: Supplementary file 1 [file ijms-22-04032-s001.pdf]

MDPI

Article

Supplementary Materials: Glass and Jamming Rheology in Soft Particles made of PNIPAM and polyacrylic acid

Silvia Franco [1,2], Elena Buratti [2], Valentina Nigro [2,3], Emanuela Zaccarelli [2,4], Barbara Ruzicka* [2,4] and Roberta Angelini* [2,4]

1 Dipartimento di Scienze di Base e Applicate per l'Ingegneria (SBAI), Sapienza Università di Roma, 00185 Roma, Italy;
2 Istituto dei Sistemi Complessi del Consiglio Nazionale delle Ricerche (ISC-CNR), Sede Sapienza, 00185 Roma, Italy;
3 ENEA C.R. Frascati, FSN-TECFIS-MNF Photonics Micro and Nanostructures Laboratory, 00044 Frascati, Rome, Italy;
4 Dipartimento di Fisica, Sapienza Università di Roma, 00185 Roma, Italy;
* Correspondence: roberta.angelini@cnr.it, barbara.ruzicka@cnr.it

Citation: Franco, S.; Buratti, E.; Nigro, V.; Zaccarelli, E.; Ruzicka, B.; Angelini, R. Supplementary Materials: Glass and Jamming Rheology in Soft Particles made of PNIPAM and polyacrylic acid. *Int. J. Mol. Sci.* **2021**, *1*, 0. https://dx.doi.org/10.3390/ijms1010000

Received:
Accepted:
Published:

Publisher's Note: MDPI stays neutral with regard to jurisdictional claims in published maps and institutional affiliations.

Copyright: © 2021 by the authors. Submitted to *Int. J. Mol. Sci.* for possible open access publication under the terms and conditions of the Creative Commons Attribution (CC BY) license (https://creativecommons.org/licenses/by/4.0/).

Keywords: rheology; microgel; shear-thinning; glass; jamming; soft colloids; PNIPAM

1. Parameters from the Cross model

Data at low concentrations $C_w \leq 0.6\%$, in the shear thinning fluid regime, are well described by the Cross model:

$$\sigma(\dot{\gamma}) = \dot{\gamma}\left[\eta_\infty + \frac{\eta_0 - \eta_\infty}{1 + (\tau_C\dot{\gamma})^m}\right], \quad (1)$$

where η_0 and η_∞ are the zero and infinite shear rate limiting viscosities respectively, m a power exponent, τ_C the relaxation time of the system that marks the onset of shear thinning [1] and its inverse $\dot{\gamma}_c$ represents an intermediate critical shear rate. In Figure S1 all the fit parameters from Cross model are reported for microgel with C_{PAAc}=24.6% at two temperatures, T=298 K and T=311 K, below and above the volume phase transition (VPT), respectively.

2. Comparison between Cross e Carreau-Yasuda models at low concentrations

The same data are also well described by the Carreau-Yasuda model:

$$\sigma(\dot{\gamma}) = \dot{\gamma}\left[\eta_\infty + (\eta_0 - \eta_\infty)\left(1 + (\tau_{CY}\dot{\gamma})^a\right)^b\right]. \quad (2)$$

The two models overlap if b=-1, a=m and τ_{CY}=τ_C. A comparison between the parameters obtained from the two models is reported in Figure S2 as a function of C_w at T=311 K showing a perfect agreement.

3. Parameters from the Herschel Bulkley model

Data at higher concentrations $0.7 \leq C_w \leq 5.0\%$, in the glass and jammed state, when a yield stress appears, are well described by the Herschel Bulkley model model:

$$\sigma(\dot{\gamma}) = \sigma_y + k\dot{\gamma}^u, \quad (3)$$

where σ_y is the yield stress, for $\sigma < \sigma_y$ there is no flow and the system behaves as a solid, for $\sigma > \sigma_y$ instead it flows, k is named "consistency" index and u is the flow index that defines the non-Newtonian behaviour, u<1 is typical of shear thinning fluids and u>1

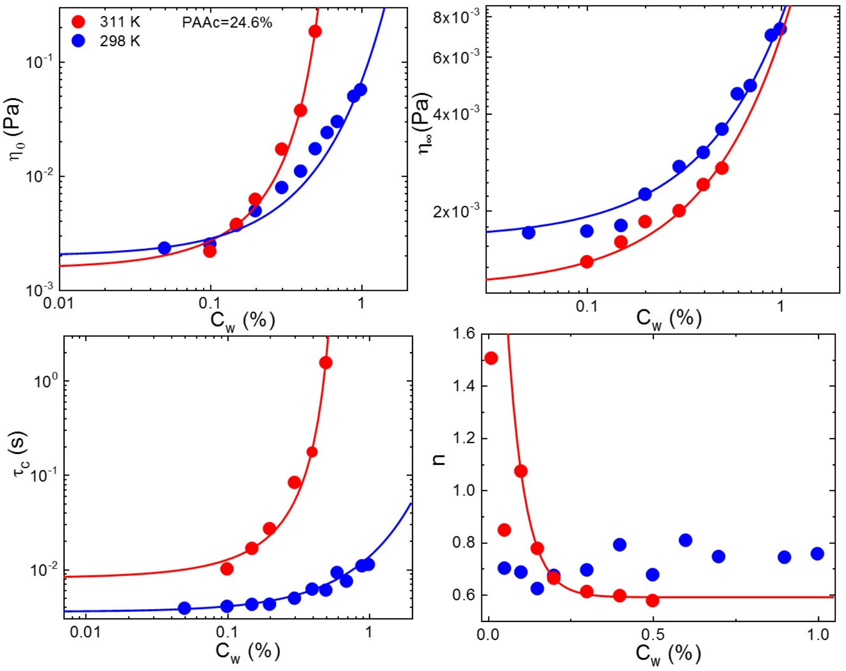

Figure S1. Fit parameters (a) η_0 (b) η_∞ (c) τ_C and (d) n, obtained from the Cross equation of IPN microgels with C_{PAAc}=24.6% at T=298 K (blue symbols) and T=311 K (red symbols).

defines systems characterized by shear thickening [2–4]. Moreover a characteristic shear rate $\dot{\gamma}_c$ can be obtained from the above parameters:

$$\dot{\gamma}_c = \left(\frac{\sigma_y}{k}\right)^{1/n}. \tag{4}$$

Fit parameters from the Herschel Bulkley model at T=311 K are reported in Figure S3 versus C_w.

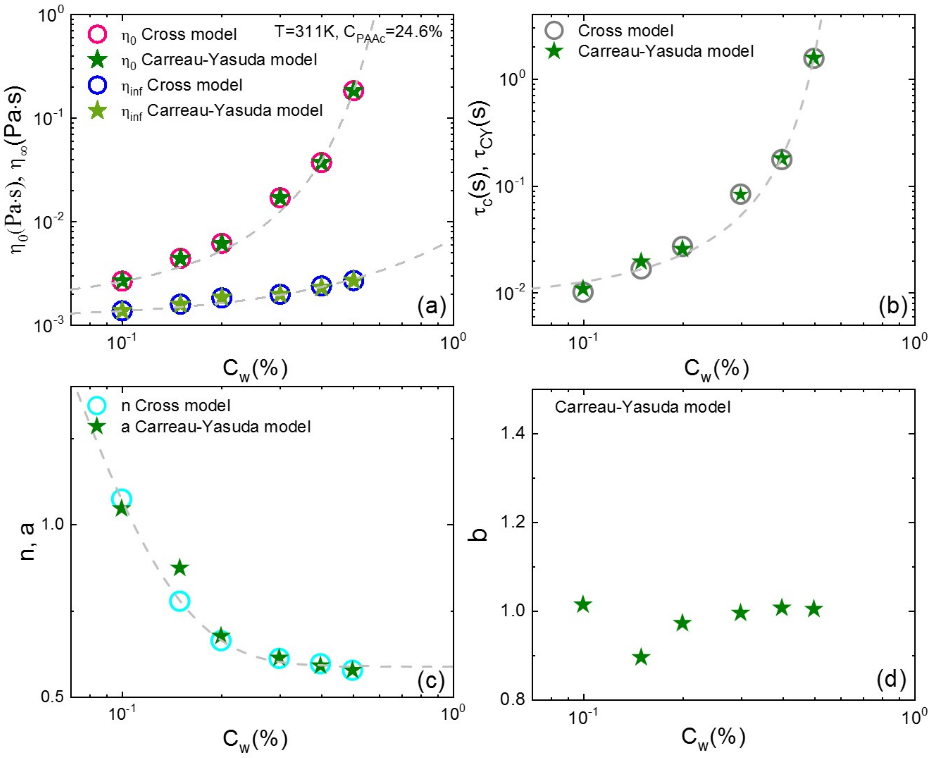

Figure S2. Comparison between the fit parameters (a) η_0, η_∞, (b) τ_C, τ_{CY} (c) n, a and (d) b, obtained from the Cross and Carreau-Yasuda models at T=311 K.

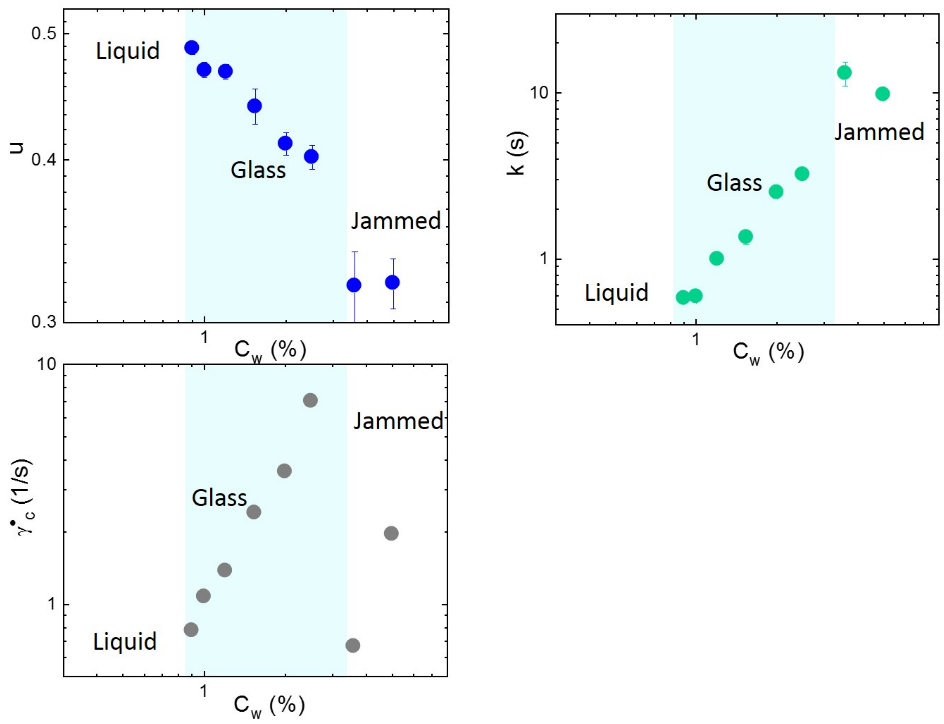

Figure S3. Fit parameters (a) u (b) k and (c) $\dot{\gamma}_c$, obtained from the Herschel Bulkley equation of IPN microgels with PAAc=24.6% as a function of concentration at T=311 K.

References

1. Pellet, C.; Cloitre, M. The glass and jamming transitions of soft polyelectrolyte microgel suspensions. *Soft Matter* **2016**, *12*, 3710–3720.
2. Ghosh, A.; Chaudhary, G.; Kang, J.G.; Braun, P.V.; Ewoldt, R.H.; Schweizer, K.S. Linear and nonlinear rheology and structural relaxation in dense glassy and jammed soft repulsive pNIPAM microgel suspensions. *Soft Matter* **2019**, *15*, 1038–1052.
3. Bonn, D.; Denn, M.M.; Berthier, L.; Divoux, T.; Manneville, S. Yield stress materials in soft condensed matter. *Phys. Rev. Lett.* **2017**, *89*, 1035005–035040.
4. Mueller, S.; W., E.; Llewellin.; Mader, H.M. The rheology of suspensions of solid particles. *The Royal Society* **2010**, *466*, 1201–1228.
